# Dicyanoacetylene (NC_4_N) Formation in the CN + Cyanoacetylene (HC_3_N) Reaction: A Combined Crossed-Molecular Beams and Theoretical Study

**DOI:** 10.1021/acsearthspacechem.5c00154

**Published:** 2025-07-31

**Authors:** Emília Valença Ferreira de Aragão, Pengxiao Liang, Luca Mancini, Gianmarco Vanuzzo, Giacomo Pannacci, Noelia Faginas-Lago, Piergiorgio Casavecchia, Marzio Rosi, Nadia Balucani

**Affiliations:** † Dipartimento di Chimica, Biologia e Biotecnologie, 9309 Università degli Studi di Perugia, Via Elce di Sotto, 8, Perugia 06123, Italy; ‡ Master-Tec S.r.l, Via Gerardo Dottori, 94, Perugia 06132, Italy; ∥ Dipartimento di Ingegneria Civile ed Ambientale, 9309 Università degli Studi di Perugia, Via G. Duranti, Perugia 06123, Italy

**Keywords:** dicyanoacetylene;, cyanopolyynes;, crossed-molecular beams;, cyano radicals

## Abstract

Unsaturated nitriles are significant in prebiotic and astrochemistry. Dicyanoacetylene, in particular, is a possible precursor of uracil and was previously detected in Titan’s atmosphere. Its null dipole moment hindered detection through rotational spectroscopy in interstellar clouds, and it escaped identification until recently, when its protonated form NC_4_NH^+^ was finally detected toward the Taurus molecular cloud (TMC-1) (Agúndez et al., Astronom. Astrophys. **2023**, 669, L1). Given the low-temperature conditions of both Titan and TMC-1, a facile formation route must be available. Low-temperature kinetics experiments and theoretical characterization of the entrance channel demonstrated that the CN + HC_3_N reaction is a compelling candidate for NC_4_N formation in cold clouds. Here, we report on a combined crossed-molecular beams (CMB) and theoretical study of the reaction mechanism up to product formation, demonstrating that NC_4_N + H is the sole open channel from low to high temperatures (collision energies). Indeed, unlike other CN reactions, the formation of the isocyano isomer (3-isocyano-2-propynenitrile) was not seen to occur at the high collision energy (44.8 kJ/mol) of the CMB experiment. Preliminary calculations on the related CN + HC_5_N reaction indicate that the reaction channel leading to NC_6_N + H is exothermic and occurs via submerged transition states. We therefore expect it to be fast and that the mechanism is generalizable to the entire family of CN +cyanopolyyne reactions. Furthermore, we derive some properties of the related reactions C_2_H + CNCN (isocyanogen) and CN + HCCNC (isocyanoacetylene): the C_2_H + CNCN reaction leads to the formation of HC_3_N + CN, and the main channel of the CN + HCCNC reaction also leads to CN + HC_3_N. This last reaction efficiently converts isocyanoacetylene and, by extension, any isocyanopolyyne into their cyano counterparts without a net loss of cyano radicals. Finally, we also characterized the entrance channel of the reaction C_2_H + NC_4_N and verified that the addition of C_2_H to all possible sites of NC_4_N is characterized by a significant entrance barrier, thus confirming that, once formed, dicyanoacetylene terminates the growth of cyanopolyynes via the sequence of steps involving polyynes, cyanopolyynes, and C_2_H/CN radicals.

## Graphical abstract

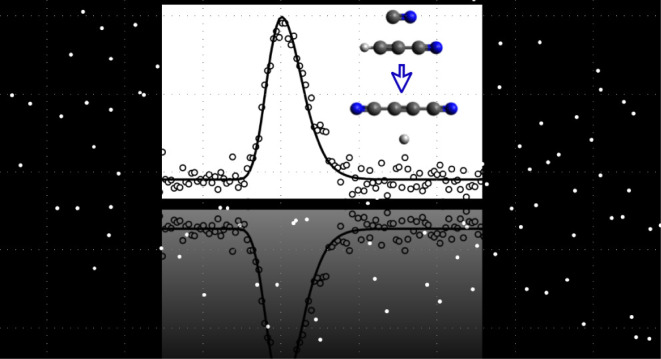

## 1 Introduction

Approximately 15–20% of all species identified in the interstellar medium (ISM) are nitriles, a family of N-bearing organic compounds characterized by a cyano (—C≡N) group. Unsaturated nitriles (including vinylcyanoacetylene, cyanoacetyleneallene or aromatic cyanonaphthalene, cyanoacenaphthylene, cyanopyrene, and cyanocoronene) rank among the most complex individual interstellar organic molecules identified to date.^1–6^ The detection of these complex nitriles was accomplished toward a cold molecular cloud, the Taurus Molecular Cloud (TMC-1), which seems to be particularly rich in nitriles. Furthermore, nitriles have been detected on Titan^7–9^ as well as in star-forming regions (high-mass and low-mass),^10–18^ protoplanetary disks,^19–21^ circumstellar envelopes (CSE) of asymptotic giant branch (AGB) stars^22–28^ in photodissociation regions (PDR),^29,30^ and cometary comae.^31–34^ Their presence in exoplanets has also been speculated.^35^ Given their significant prebiotic potential and the conjectured role they may have played in the chemistry that led to the emergence of life,^36,37^ nitriles have garnered considerable attention, prompting extensive investigation into their formation pathways and astrochemical/photochemical models (see, for instance, refs. 12, 18, 38–57 ).

More recently, given the increased sensitivity of radio telescopes, the first detection of interstellar dinitriles (molecules containing two cyano groups) has become accessible. The first dinitriles identified were maleonitrile (Z-but-2-enedinitrile or cis-1,2-dicyanoethylene) and malononitrile (propanedinitrile or dicyanomethane), both identified toward TMC-1.^58^ Prior to this, isocyanogen (C≡N—C≡N) and protonated cyanogen had been detected,^59,60^ while attempts to detect larger saturated dinitriles^61,62^ and dicyanobenzene^63^ failed. Furthermore, and of relevance to the present work, the detection of dicyanoacetylene (2-butynedinitrile; N≡C—C≡C—C≡N, henceforth indicated by NC_4_N) in its protonated form NC_4_NH^+^ (an otherwise nonobservable apolar dinitrile) finally confirmed its presence,^64^ which, together with that of larger dicyanopolyynes, had been postulated long ago.^65,66^ NC_4_N is considered a precursor of uracil via its hydrolysis to acetylenedicarboxylic acid and subsequent reaction with urea.^67^ Furthermore, in a recent study, a new α-cytidine derivative was successfully synthesized from the reaction of ribose aminooxazoline and dicyanoacetylene.^68^ Therefore, it is particularly interesting to understand the formation routes of dicyanoacetylene in space and its possible connection with the emergence of life on Earth.

Both cyanogen (C_2_N_2_) and dicyanoacetylene (in the form of ice) had already been identified in the atmosphere of Titan, the massive moon of Saturn.^8,9,69^ Their detection prompted speculation on their possible formation routes long ago.^47^ In the case of dicyanoacetylene, the first suggested formation route was the reaction HCCN + HCCN → NCCCCN + H_2_, but it was later speculated that HCCN (formed by the reaction N­(^2^D) + C_2_H_2_)^70^ reacts rather with H or CH_3_ radicals.^71^ The other NC_4_N formation route suggested by Yung^47^ was the reaction

1 $$\mathrm{CN}+{\mathrm{HC}}_{3}N\to {\mathrm{NC}}_{4}N+H$$

in analogy with the related reaction

2 $$\mathrm{CN}+C_{2}H_{2}\to {\mathrm{HC}}_{3}N+H$$

A rate coefficient of 10^–11^ cm^3^·s^–1^ was adopted by Yung in his upgraded photochemical model of the atmosphere of Titan, following the indications of experimental measurements by Halpern et al., who later published their work reporting a value of (1.7 ± 0.08) × 10^–11^ cm^3^ s^–1^ at room temperature.^72^ A barrier of 1.5 kcal mol^–1^ (6.3 kJ mol^–1^) was suggested upon comparison with the rate coefficients of other CN reactions with unsaturated hydrocarbons. This room-temperature data were used by Faure et al.^73^ to estimate, using a semiempirical model coupled to the capture theory, the *k*(*T*) value in the temperature range from 10 to 295 K. Finally, recent kinetic measurements by using the CRESU technique derived the *k*(*T*) below room temperature, reaching a value as low as 22 K.^74^ The reaction rate coefficient was seen to increase with decreasing temperature, which is incompatible with the significant entrance barrier inferred by Halpern et al.^72^ However, the rate coefficients at low temperatures are smaller than those predicted by Faure et al.^73^ In addition to the experimental values, Cheikh Sid Ely et al.^74^ also characterized the entrance channel of the potential energy surface (PES) of reaction (1) and were able to reproduce the trend of the experimental rate coefficient as a function of temperature by employing a two-transition-state (2TS) model.^75^ A similar trend was also obtained by Valença Ferreira de Aragão et al.,^76^ who used a semiempirical analysis of the long-range interactions based on an improved Lennard–Jones potential. Both theoretical studies focused only on the entrance channel, and the full PES up to the products was not derived. In addition, both the kinetic experiments by Cheikh Sid Ely et al.^74^ and Halpern et al.^72^ followed only the decay rate of the reactants and, therefore, could not provide information on the reaction products.

Some information on the global PES of reaction (1) can be derived from the work of Petrie and Osamura, which focuses on the reaction between C_3_N and HNC as the main formation pathway of NC_4_N in the atmosphere of Titan.^77^ The products of that reaction include, among others, cyanoacetylene and cyano radicals in one channel and dicyanoacetylene and atomic hydrogen in another channel. In the PES by Petrie and Osamura, there is a pathway connecting these products, featuring one intermediate and a transition state. However, the full potential energy surface of the CN + HC_3_N reaction has yet to be reported, and the CN addition on the N-side has never been considered before. This last aspect could be relevant for high-energy environments since, in the case of the related CN reactions with acetylene and ethylene, crossed-molecular beam (CMB) experiments provided evidence that the channels leading to the isocyano isomeric products (isocyanoacetylene and isocyanoethylene) become open at high collision energies.^78–80^

In conclusion, although dicyanoacetylene + H has always been considered the only open reaction channel, a comprehensive study of the PES of the title reaction is still lacking, as is experimental evidence of the nature of the reaction products. In this work, we report the results of a CMB experiment that demonstrates that dicyanoacetylene + H is the sole open reaction channel up to high collision energies (and therefore high temperatures). The CMB results are complemented by the derivation of the complete reactive potential energy surface, in which the CN addition on the N-side is also considered, to verify whether 3-isocyano-2-propynenitrile (N≡C—C≡C—N≡C, henceforth indicated by the simplified formula CNC_3_N) can be formed at high collision energies. The formation of isocyano isomers could be relevant in high-temperature environments, such as flames or the CSE of AGB stars and photodissociation regions, where isonitrile species have been identified.^26,27,30^

## 2 Methods

### 2.1 The Crossed-Molecular Beam Technique with Mass Spectrometric Detection

The crossed-molecular beam (CMB) apparatus, equipped with universal quadrupole mass spectrometric (QMS) detection and time-of-flight (TOF) analysis, used in the present study, has been described in detail elsewhere.^81,82^ Therefore, only a concise description is provided here. Two collimated, continuous supersonic beams of the reactants are crossed at 90° in a large scattering chamber maintained at 10^–5^ Pa to ensure single-collision conditions. Reactants and products were detected by a triply differentially pumped ultrahigh-vacuum detection system, maintained at a pressure of less than 10^–9^ Pa during operation and equipped with an electron impact ionizer, a quadrupole mass filter, and a Daly detector.^83^ The entire detector unit can be rotated in the collision plane around an axis that passes through the collision center.

The product angular distribution, N­(Θ), that is, the intensity of the products as a function of the laboratory (LAB) scattering angle Θ, was obtained by taking at least five scans per angle, with a counting time of 100 s and by modulating the cyanoacetylene beam with a tuning fork chopper at a frequency of 160 Hz for background subtraction. Velocity distributions of the reactants were determined by measuring in-axis single-shot time-of-flight (TOF) spectra. Product TOF distributions, N­(Θ,t), were obtained at selected LAB angles by using the TOF pseudorandom chopping technique, with the pseudorandom wheel (containing four identical sequences of 127 open/closed elements) spinning in front of the entrance of the detector at 328.1 Hz (corresponding to a dwell time of 6 μs/channel).

The beam of CN radicals was generated by the high-pressure radio frequency (RF) discharge beam source successfully used in our laboratory over a number of years to generate intense supersonic beams of atoms and radicals.^84–87^ More specifically, the CN beam was generated by expanding a gas mixture with the composition CO_2_(0.8%)/N_2_(2.5%)/He at 90 hPa (quartz nozzle diameter was 0.45 μm, and RF power 300 W); the CN beam had a peak velocity of 2184 m/s and a speed ratio of 5.5. Typically, in CMB experiments utilizing supersonic beams, the internal energy content of the reactants is minimal due to the extensive cooling of internal degrees of freedom during expansion. Nevertheless, in this case, the beam of CN radicals is formed chemically in situ by the transient species produced in the discharge plasma starting from CO_2_/N_2_/He and the radicals maintain a large internal energy content. By using a laser-induced fluorescence (LIF) characterization,^85^ we verified that the cooling of the internal excitation is incomplete and that the CN radicals are ro-vibrationally excited. More specifically, the CN vibrational distribution is consistent with a vibrational temperature of 6500 K, while the rotational distribution is bimodal, with two peaks around *N* = 6 and *N* = 39–44.^85^

Cyanoacetylene (HC_3_N) was synthesized by following the two-stage procedure described by Miller and Lemmon^88^ (see also Liang et al.).^89,90^ The HC_3_N white crystals were stored in a glass vial immersed in a 261 K constant-temperature bath to prevent polymerization and maintain a stable equilibrium vapor pressure. The HC_3_N beam was produced by expanding the vapor (pressure around 64 hPa) through a 0.1 mm stainless steel nozzle. The internal energy content of the HC_3_N molecule is expected to be small because the internal degrees of freedom are cooled during the supersonic expansion. The HC_3_N beam had a peak velocity of 657 m/s and a speed ratio of 3.5.

Under these experimental conditions, the average collision energy (E_*c*_) was 44.8 kJ/mol. The center-of-mass (CM) position angle, Θ_CM_, was 30.6°.

The scattering measurements were carried out in the LAB system of coordinates, while for the physical interpretation of the scattering process, it is necessary to transform the data (angular, N­(Θ), and time-of-flight N­(Θ, t) distributions) to a coordinate frame of reference that moves with the center-of-mass (CM) of the colliding system. The relation between the LAB and CM product flux is given by I_*LAB*_(Θ,*v*) = I_*CM*_(θ,*u*) × *v* ^2^/*u* ^2^, where Θ and *v* are the LAB scattering angle and velocity, respectively, while θ and *u* are the corresponding CM quantities (see the velocity vector, or “Newton,” diagram in Figure 1). Since the mass spectrometric detector measures the number density of products, N­(Θ), rather than the flux, the actual relation between the LAB density and the CM flux is given by N_*LAB*_(Θ,*v*) = I_*CM*_(θ,*u*) × *v*/*u* ^2^. The final outcome of a reactive scattering experiment is therefore the so-called differential cross-section I_*CM*_(θ,*u*), which is commonly factorized into the product of the velocity (or translational energy) distribution, P­(*u*) (or P­(E’_*T*_)), and the angular distribution, T­(θ). Because of the finite resolution of experimental conditions, i.e., finite angular and velocity spread of the reactant beams and angular resolution of the detector, the LAB-CM transformation is not single-valued, and analysis of the laboratory data is carried out by the usual forward convolution procedure in which trial CM angular, T­(θ), and translational energy, P­(E’_*T*_), distributions are assumed, averaged, and transformed to the LAB for comparison with the experimental data until the best fit of the LAB distributions is achieved.^82^ The best-fit CM T­(θ) and P­(E’_*T*_) functions contain all of the information about the reaction dynamics.

**Figure 1:**
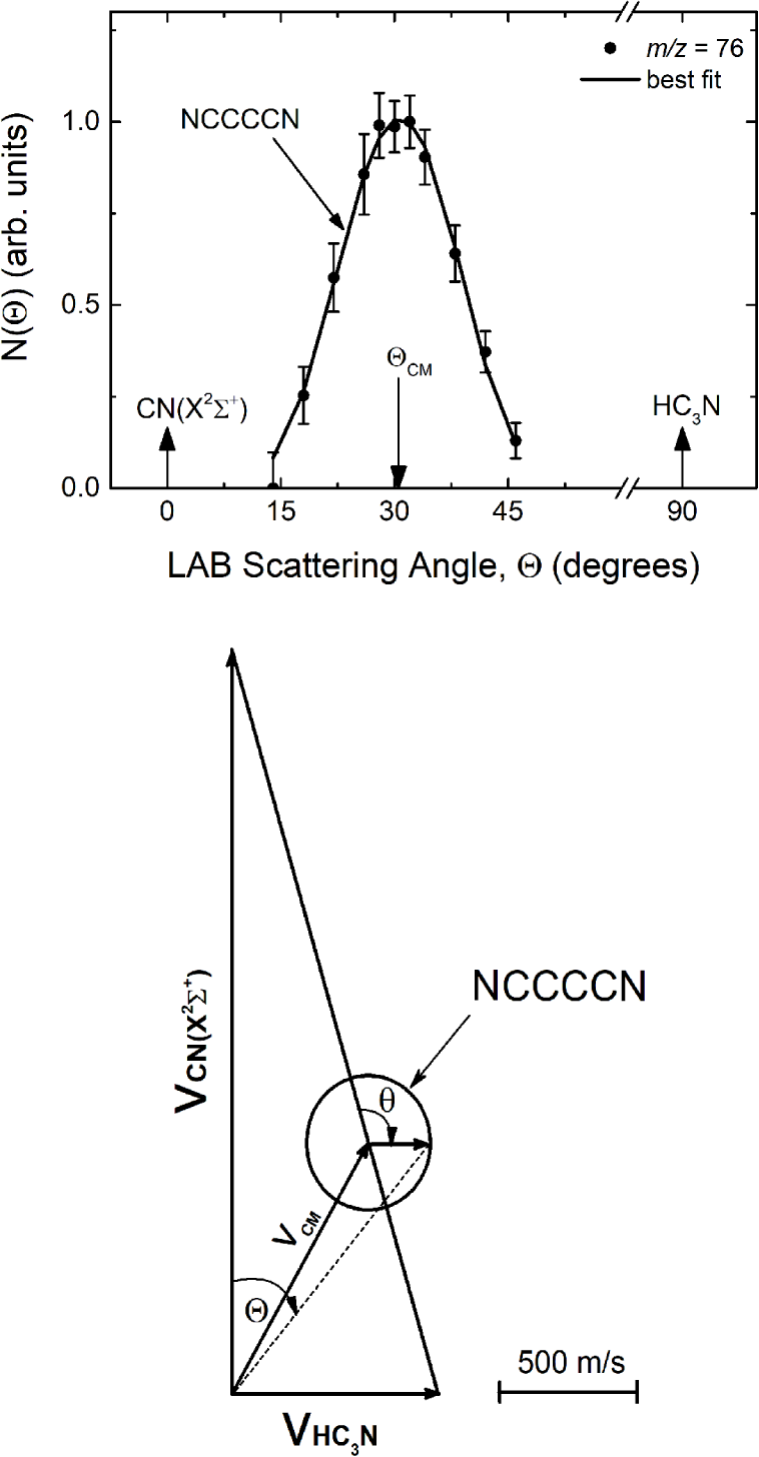
Laboratory angular distribution of the NC_4_N product at *m*/*z* = 76 from the reaction CN (X^2^Σ^+^) + HC_3_N at E_*c*_ = 44.8 kJ/mol using a 70 eV electron energy at an emission current of 2 mA and the corresponding velocity vector (Newton) diagram. The dots represent the experimental data (an average of at least 5 scans) and their standard deviation. The solid black line in the upper figure represents the calculated distribution using the best-fit CM angular and translational energy functions from Figure 3. Θ_CM_ defines the CM position angle. The circle in the Newton diagram delimits the maximum center-of-mass speed and therefore, the angular range within which the NC_4_N product can be scattered (see text). The analogous circle for CNC_3_N could not be drawn because it is too small.

### 2.2 Electronic Structure Calculations of the Reaction Potential Energy Surface

The potential energy surface of the doublet CN–HC_3_N system was investigated by searching for and optimizing the relevant stationary points and product channels. Calculations were performed adopting an unrestricted formalism using the Gaussian 09 code.^91^ Following a well-established computational scheme, the geometries of minima and saddle points were optimized using a less expensive method compared to the one employed to get more accurate energy values.^89,90,92–94^ Preliminary benchmark calculations to test the theoretical methods were presented in ref. 95.

Geometry optimization calculations were performed using density functional theory (DFT), with the Becke-3-parameter exchange and Lee–Yang–Parr correlation (B3LYP)^96,97^ in conjunction with the correlation-consistent valence polarized basis set aug-cc-pVTZ.^98^ In some cases, the structures were optimized with the hybrid meta exchange-correlation functional M06–2X^99^ in conjunction with 6–311+G­(d,p).^100,101^ Vibrational frequency analysis was used to determine the nature of stationary points: a minimum in the absence of imaginary frequencies or a saddle point if one frequency and only one frequency is imaginary. Furthermore, intrinsic reaction coordinate (IRC) calculations^102,103^ have been performed for each saddle point geometry to confirm that it corresponds to a true transition state between two minima. After optimization, the energies of the stationary points were computed with coupled-cluster CCSD­(T)^104–106^ and aug-cc-pVTZ basis set to achieve more accurate energetics. All energies presented in this paper are the CCSD­(T) energies corrected with zero-point energies from frequency calculations. The T1 diagnostic values for all species included in the minimum energy path, for both the C-side and N-side addition, support the adequacy of single-reference methods such as CCSD­(T) (see Table S1).^107^

Finally, for the two most important reaction pathways originating from the C-side and N-side addition of CN to the triple C–C bond of HC_3_N, more accurate single-point energy calculations were performed at the CCSD­(T) level, corrected with a density fitted (DF)­MP2 extrapolation to the complete basis set (CBS) and with corrections for core electron excitations. In particular, the energies were computed as

3 E(CCSD(T)/aug‐cc‐pVTZ)+E(1)+E(2)

with

$$\begin{array}{ll} E\left(1\right) & =E\left(\mathrm{CCSD}\left(T,\mathrm{core}\right)/\mathrm{cc‐pVTZ}\right)-E\left(\mathrm{CCSD}\left(T\right)/\mathrm{cc‐pVTZ}\right)\\ E\left(2\right) & =E\left(\mathrm{DF‐MP2}/\mathrm{CBS}\right)-E\left(\mathrm{DF‐MP2}/\mathrm{aug‐cc‐pVTZ}\right) \end{array}$$

and where *E*(DF-MP2/CBS) is defined as

4 $$\begin{array}{ll} E\left(\mathrm{DF‐MP2}/\mathrm{CBS}\right) & =E\left[\left(\mathrm{DF‐MP2}\right)/\mathrm{aug‐cc‐pVQZ}\right)]+\\ 0.5772\times [E\left(\mathrm{DF‐MP2}/\mathrm{aug‐cc‐pVQZ}\right)+\\ -E\left(\mathrm{DF‐MP2}/\mathrm{aug‐cc‐pVTZ}\right)] \end{array}$$

The *E*(DF-MP2/CBS) extrapolation was performed using Martin’s two-parameter scheme.^108^

## 3 Results

### 3.1 Results of the Crossed-Molecular Beam Experiments

Reactive scattering signals were recorded at *m*/*z* = 76, corresponding to the parent ion of products with the gross formula C_4_N_2_. Since the parent peak is largely dominant in the electron ionization of dicyanoacetylene[Fn fn1] and there was no gain in the signal-to-noise ratio when using the soft ionization approach,^81,109^ all the measurements were performed at *m*/*z* = 76 using an energy of 70 eV for the ionizing electrons. The product laboratory angular distribution detected at *m*/*z* = 76, together with the relevant velocity vector (“Newton”) diagram, is reported in Figure 1. The error bars (representing ± 1 standard deviation) are also shown when they exceed the size of the dots, indicating the intensity averaged over the different scans. In the Newton diagram of Figure 1, the Newton circle relative to NC_4_N scattered by the H coproduct is drawn on the assumption that all the available energy is converted into product translational energy for the reaction of CN in its ground rovibrational level (v = 0, *N* = 0). The Newton circles relative to the reactions involving the excited vibrational levels are very similar and are not shown for the sake of simplicity. The Newton circles delimit the LAB angular range within which NC_4_N can be scattered. Since in our previous experiments on the CN + C_2_H_2_ and CN + C_2_H_4_ reaction, we had experimental evidence of the formation of the isocyano products (with a small yield), we also considered the possibility that CNC_3_N could be formed in an H-displacement mechanism. The quite different enthalpy change of the two channels (see section 3.2) implies a very different extension of the Newton circles, with the angular distribution of CNC_3_N confined between Θ = 27° and 33°. No apparent features can be associated with a contribution of this kind.

The TOF spectra measured at selected angles are reported in Figure 2.

**Figure 2:**
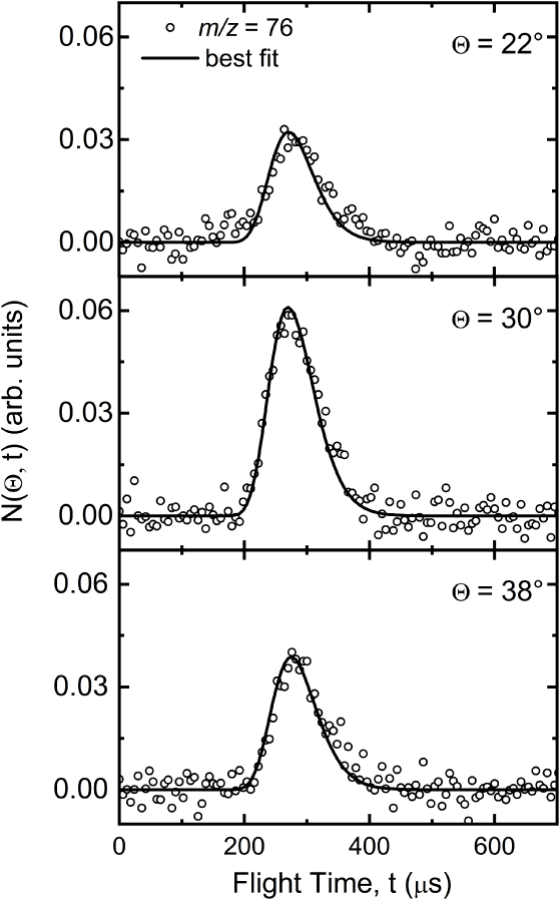
Time-of-flight (TOF) distributions of NC_4_N (*m*/*z* = 76) product detected at selected LAB scattering angles using a 70 eV electron energy. Counting times: 170 min at 22°, 120 min at 30°, and 180 min at 38°, respectively. The empty circles indicate the experimental data, while the solid black lines represent the distributions obtained with the best-fit functions shown in Figure 3.

The angular distribution extends for 30° and has a bell-shaped curve around the peak, which is located at the center-of-mass angle. The TOF distributions were recorded at Θ_CM_ and in the forward (Θ = 22°) and backward (Θ = 38°) directions. Also, in the case of TOF spectra, no features are visible that could be associated with the channel leading to CNC_3_N + H. If it were present, the TOF distribution recorded at (Θ = 30°) would show a distinct component not present in the other two TOF spectra.

The solid lines in Figures 1 and 2 represent the curves calculated with the best-fit functions reported in Figure 3. The best-fit CM angular distribution (top panel of Figure 3 is isotropic, with equal intensity from 0° to 180°. A slightly polarized T­(θ) (with T(90°) = 0.7) or sideways T­(θ) (with T(90°) = 1.3) still affords an acceptable fit of the experimental distributions (see the shaded areas in Figure 3). The T­(θ) shape is consistent with the formation of a bound intermediate with a lifetime longer than its rotational period.

**Figure 3:**
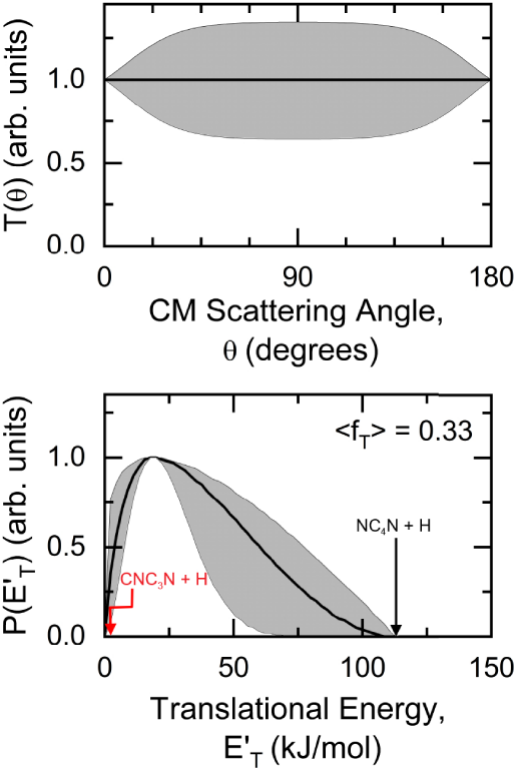
Best-fit CM product angular distribution (top) and translational energy distribution (bottom) for the NC_4_N + H channel. In the bottom panel, the black arrow indicates the total available energy for NC_4_N + H when considering the reaction of CN in its ground rovibrational level. The red arrow, instead, indicates the total available energy for the CNC_3_N + H isomeric channel. The shaded areas indicate the error bars for the best-fit CM functions.

Regarding the best-fit product translational distribution, P­(E’_T_), shown in Figure 3 (bottom panel), we observe that the peak is centered around 19 kJ/mol, while the tail extends to approximately 110 kJ/mol. The peak position suggests the presence of a small exit barrier. We recall that in this kind of experiment, the fit of both angular and TOF distributions is very sensitive to the rise and the peak position, while it is less sensitive to the tail of the P­(E’_T_) (in this case, from E’_T_ = 55 kJ/mol). Nonetheless, it is quite clear that the extra amount of energy carried by the CN vibrational excitation (which is quite sizable, being Δ*V*_1–0_ = 24.4 kJ/mol, Δ*V*_2–0_ = 48.6 kJ/mol, Δ*V*_3–0_ = 72.4 kJ/mol) is not necessary to fit the experimental distributions (the indicated black arrow in Figure 3 refers to the total available energy for the reaction channel leading to NC_4_N + H and involving CN in v = 0). The average product translational energy, defined as $<{E^{\prime }}_{T}>=\Sigma P\left({E^{\prime }}_{T}\right)\times {E^{\prime }}_{T}/\Sigma P\left({E^{\prime }}_{T}\right)$ , is about 37 kJ/mol. We recall that the total available energy (E_Tot_) is the sum of the collision energy, internal energy of the reactants (E_int_), and the reaction exothermicity (− ${\Delta H}_{0}^{◦}$ ). We considered here the value of ${\Delta H}_{0}^{◦}$ that we calculated at the highest level of theory, that is, −64.4 kJ/mol. When considering E_Tot_ for the reaction involving CN­(v = 0) and leading to the products NC_4_N + H, the average fraction of total available energy released into translation is 0.33, a typical value considering the energy profile of the PES as far as the pathway leading to NC_4_N + H is concerned.

As noted, the additional energy from the rovibrational excitation of CN does not need to be considered in the experimental distributions. This implies that the reaction is vibrationally adiabatic (the initial vibrational excitation of the CN reactant is retained as such in the final products and is not converted into translational energy of the products), as already observed in related reactions, including CN + C_2_H_2_, CN + CH_3_CCH,^85^ and CN + C_2_H_4_.^78,79^

Concerning the possible occurrence of the channel leading to the isomer CNC_3_N, we note that, according to our theoretical calculations (see the next section), the ${\Delta H}_{0}^{◦}$ is +42.3 kJ/mol at our best level of theory. Therefore, considering the E_*c*_ of 44.8 kJ/mol, this channel is energetically permitted for only 2.5 kJ/mol. If a channel with this small amount of E_Tot_ were open, even with a very low yield, we would see a strong signal associated with it because of the large enhancement produced by the transformation Jacobian, which strongly amplifies the slow products. A centroid distribution around Θ_CM_ would be clearly visible. In Figure S1, the simulated angular and TOF distributions when using a P­(E’_T_) compatible with this channel (also reported in Figure S1) are shown as an example. The presence of excited rovibrational levels of CN is not expected to alter this since in the CN + C_2_H_4_ reaction, where we were able to disentangle the contribution of the isocyanoethylene channel, there was again no indication that the internal energy content of CN was converted into product translational energy. According to our PES calculations, an exit barrier of 64.9 kJ/mol is present above the energy of the reactants for this channel. Therefore, excited CN radicals in v = 2 and v = 3 do have enough energy to overcome the barrier. However, it is well known that vibrational excitation of the CN radical is not suitable for promoting this less favorable reaction channel (see, for instance, kinetic experiments on the related systems CN + C_2_H_2_, CN + C_2_H_6_, and CN + CH_4_).^110,111^

### 3.2 Electronic Structure Calculations

The structures (interatomic distances, bond angles, and dihedral angles) of the reactants, possible products, and stationary points of the PES are shown in Figures 4–6. Most geometric configurations have been optimized at the B3LYP/aug-cc-pVTZ level, with the exception of the van der Waals complex (vdW) in the entrance channel and the transition state (TS) that connects vdW with the addition intermediate INT1, which have been computed at M062*X*/6–311+G (d,p). Figures 7 and 8 depict the two portions of the PES that originate when the CN radical interacts with cyanoacetylene on the C-side and the N-side, respectively. All reported energy values are at the CCSD (T)/aug-cc-pVTZ level and include ZPE. We have characterized the lowest energy paths (in red in Figures 7 and 8) for the two portions of the PES, also at the higher CBS level, to provide a better evaluation of the energy values as they are critical to interpreting the outcome of the CMB experiments.

**Figure 4:**
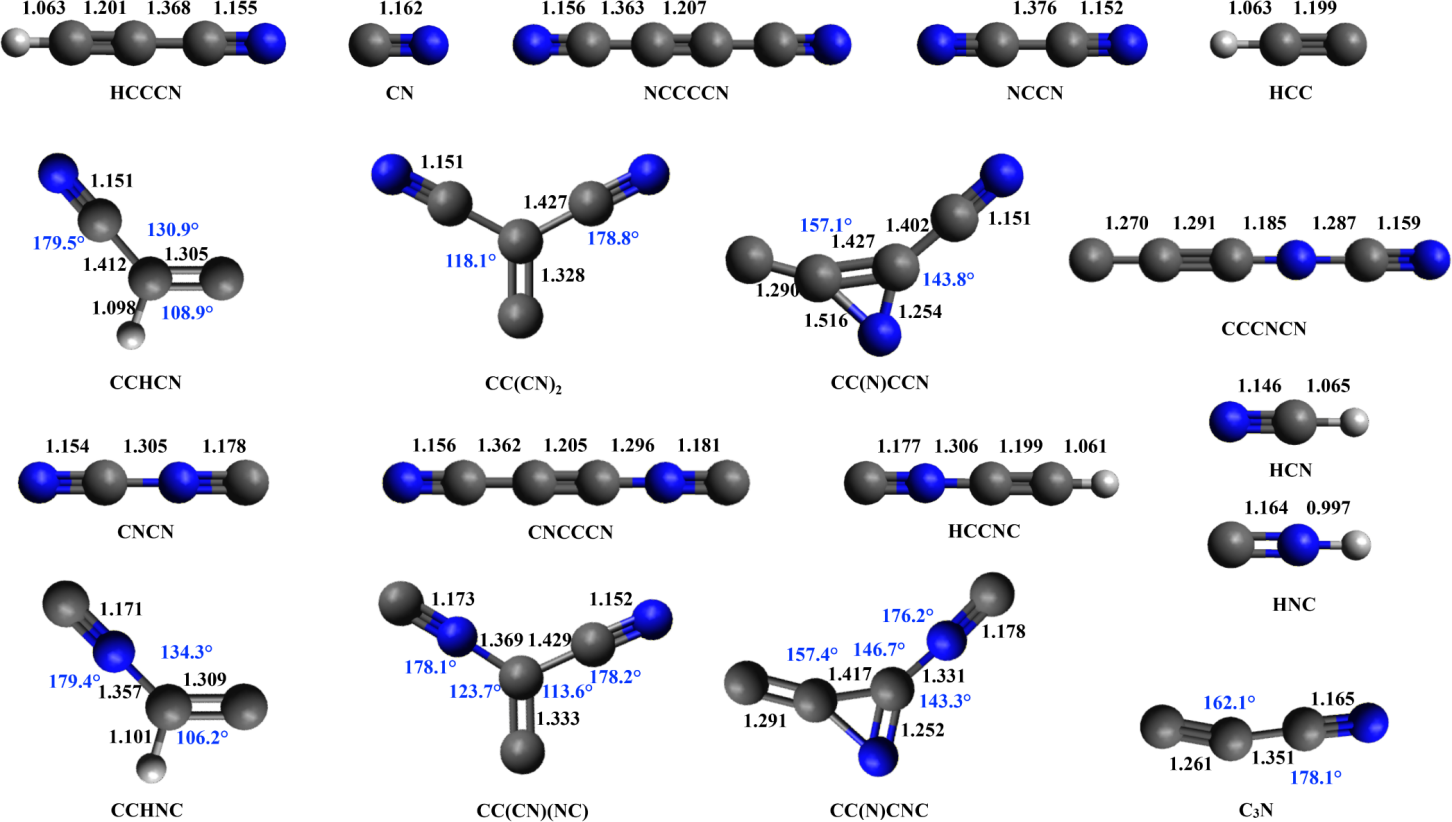
Geometries of reactants and products were optimized at the B3LYP/aug-cc-pVTZ level. Bond lengths (in Å) are displayed in black, and bond angles (in degrees) are in blue. Carbon atoms are represented in gray, hydrogen atoms in white, and nitrogen atoms in blue.

**5:**
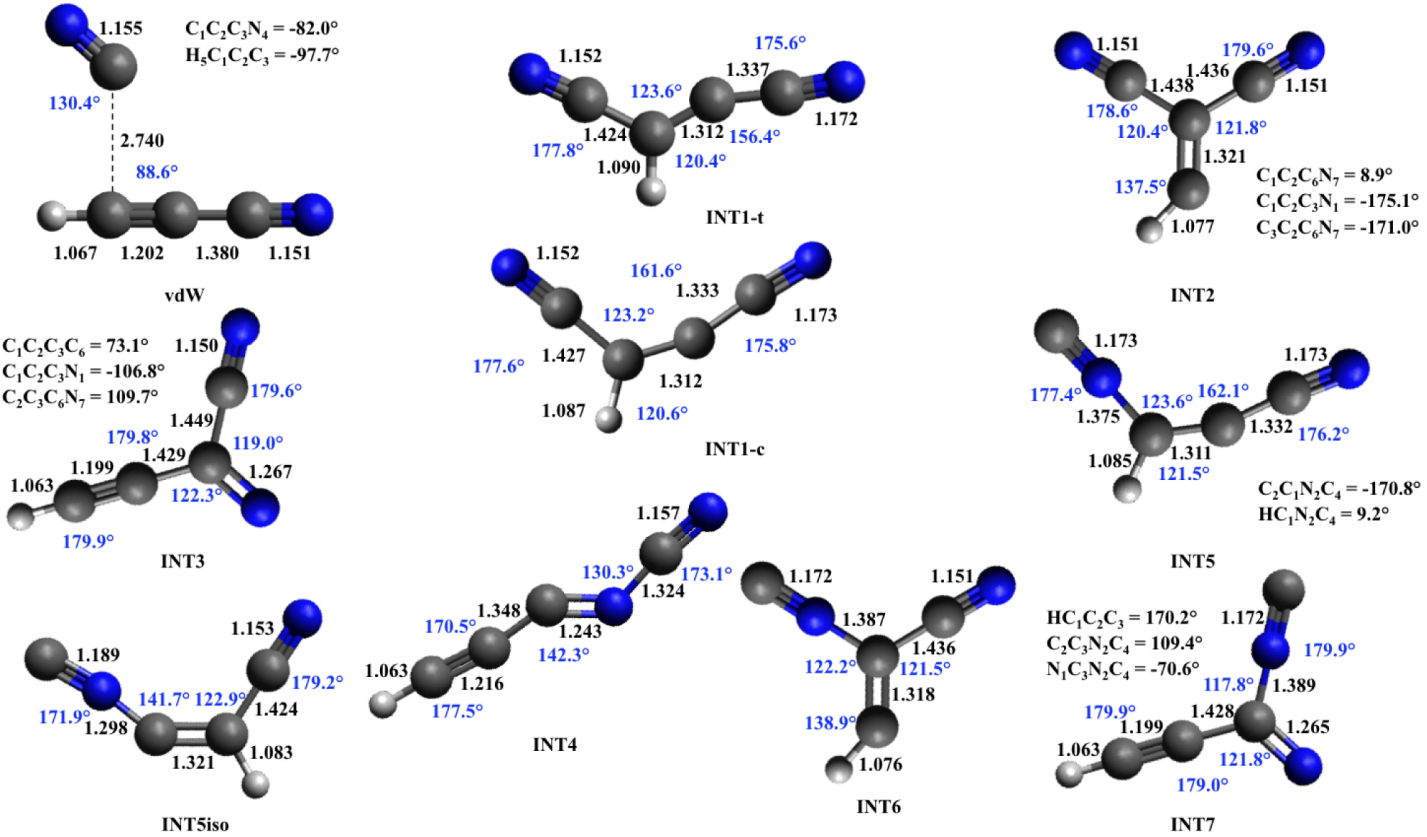
Geometries of intermediates optimized at the B3LYP/aug-cc-pVTZ level. The vdW geometry was optimized at the M062*X*/6–311+G (d,p) level. The units of distance and angle and the color scheme are the same as in Figure 4.

**Figure 6:**
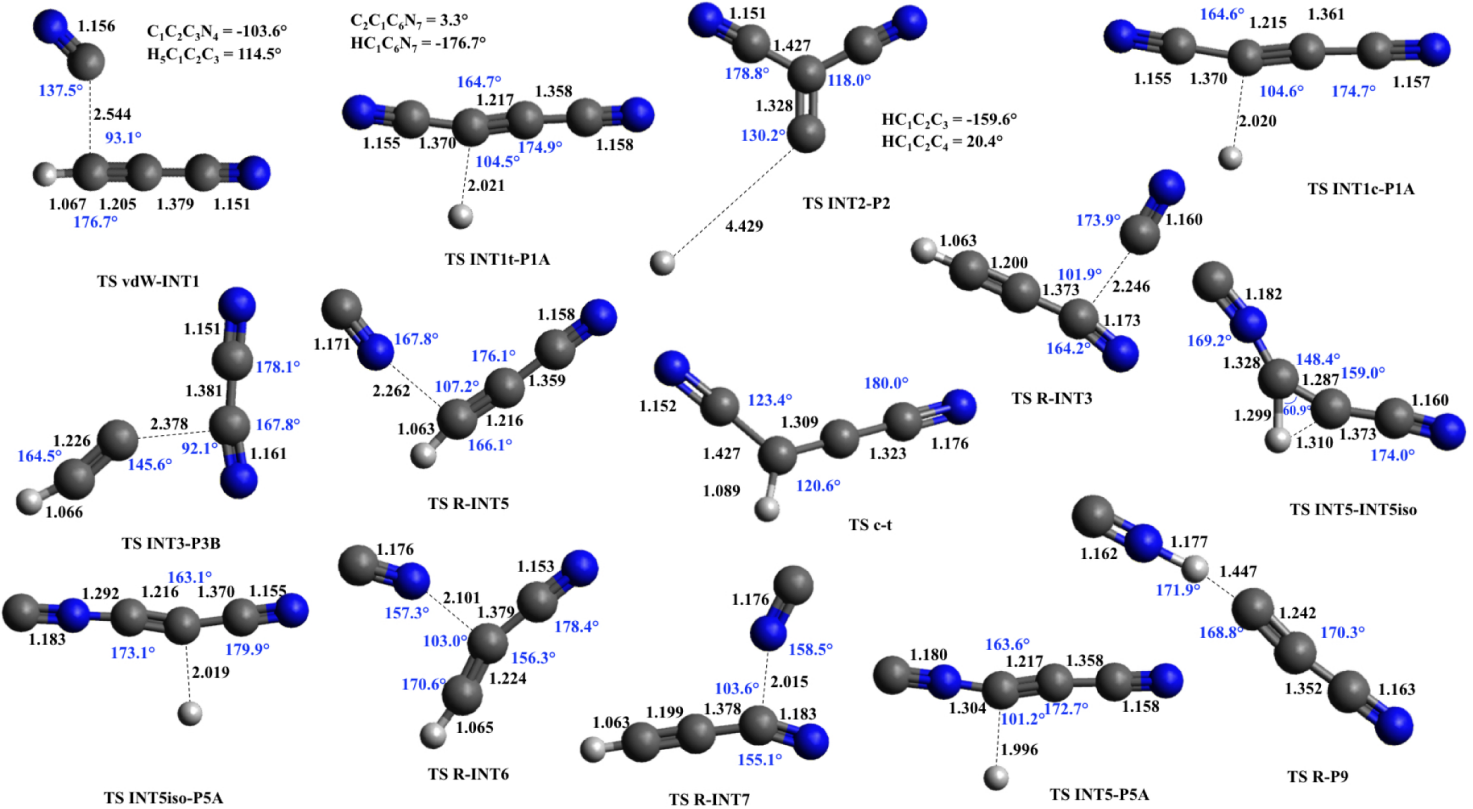
Geometries of saddle points optimized at the B3LYP/aug-cc-pVTZ level. TS vdW-INT1 was optimized at the M062*X*/6–311+G­(d,p) level. The units of distance and angle and the color scheme are the same as in Figure 4.

**Figure 7:**
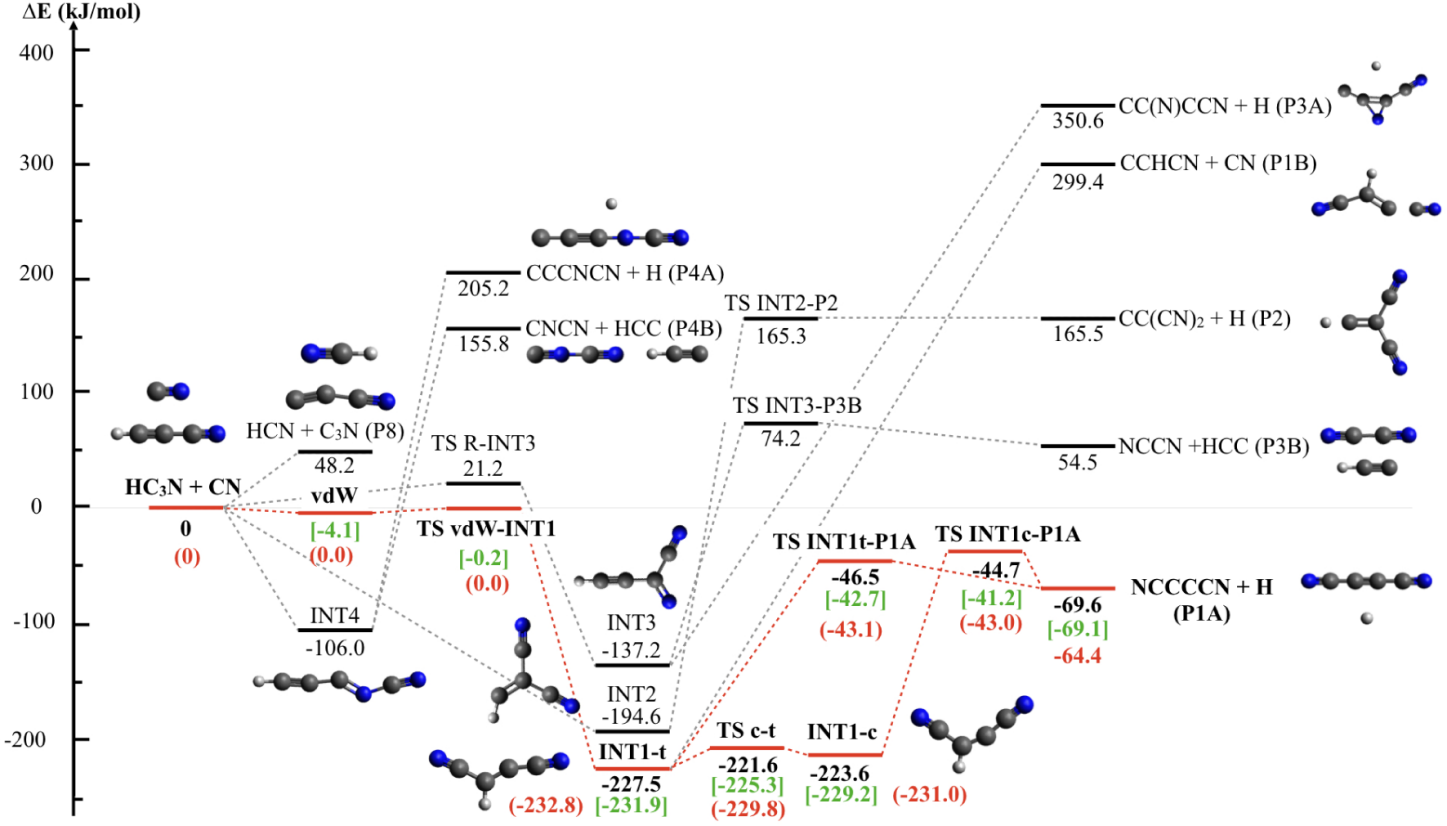
Portion of the potential energy surface with products resulting from the addition of the C-side of the cyano radical. Energies (kJ/mol) in black were computed at the CCSD­(T)//B3LYP/aug-cc-pVTZ level. Energies (in kJ/mol) are in green at the CCSD­(T)//M062*X*/6–311+G­(d,p) level. Shown in red is the most favorable pathway, with the energy values calculated also at the CBS level.

**Figure 8:**
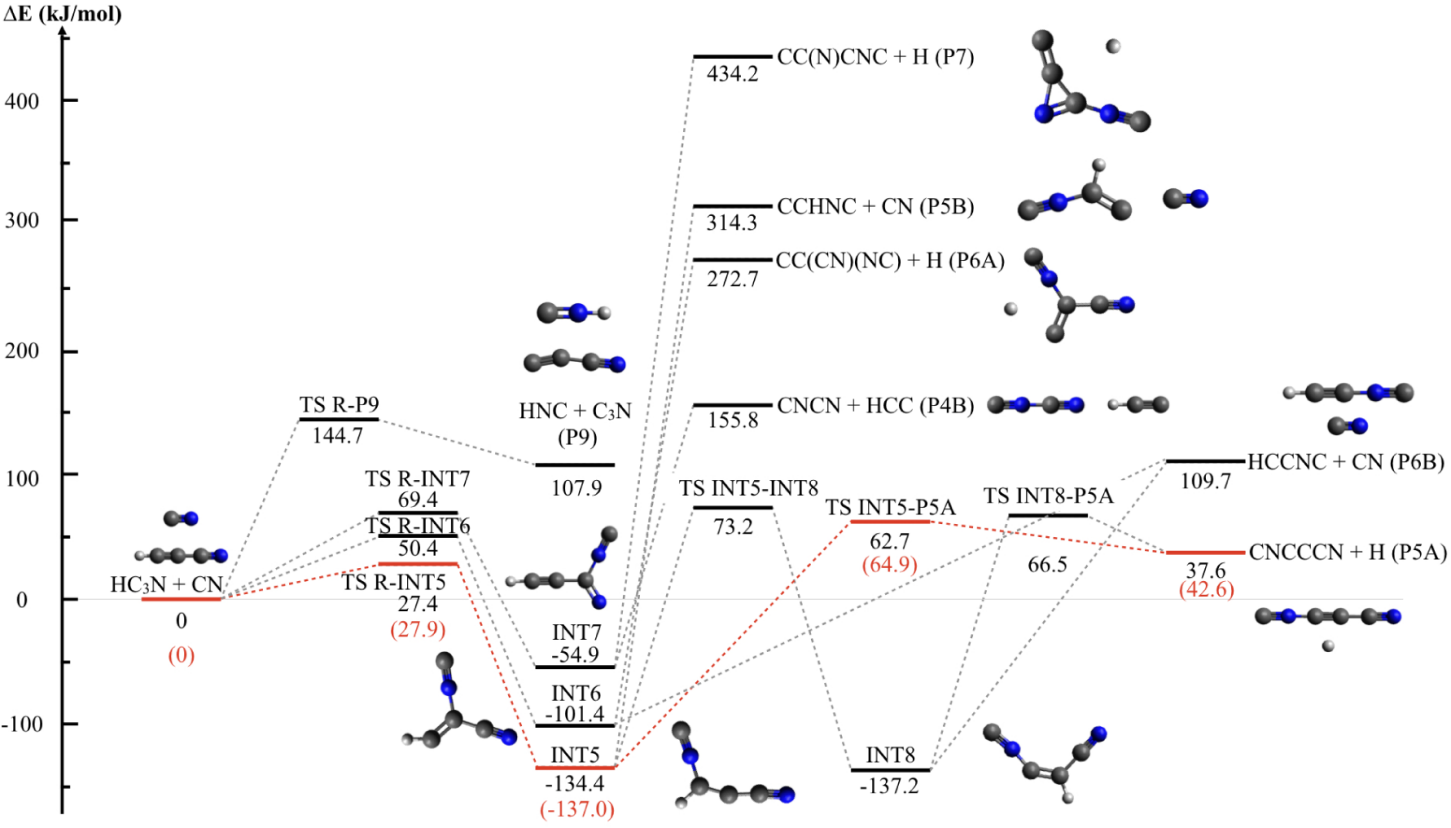
Potential energy surface with products resulting from the addition of the N-side of the cyano radical. Energies (kJ/mol) were computed at the CCSD­(T)//B3LYP/aug-cc-pVTZ level. Shown in red is the most favorable pathway, with the energy values calculated also at the CBS level.

The interaction of the cyano radical with cyanoacetylene, from both the C-side and N-side, can occur via: (a) the attack on the π-system of the C–C triple bond; (b) the attack on the π-system of the C–N triple bond; and (c) H-abstraction. In the case of the interaction from the C-side, addition to the lone pair of N of the HC_3_N cyano group is also possible.

A concise description of the reaction mechanism for all of the reaction channels is provided below.

Addition of the cyano radical on the C-side can occur in four different ways: *(1)* The interaction with the C–C triple bond can lead to the CN addition on the unsubstituted acetylenic carbon, resulting in the formation of INT1-t that easily isomerizes to the *cis* isomer INT1-c; both isomers can undergo H-loss by overcoming a barrier (TS INT1t-P1A and TS INT1c-P1A, respectively), located at ca. 21 kJ/mol above the energy of the NC_4_N + H products. This is the only exothermic reaction channel of the entire PES. The entrance channel is characterized by the formation of a van der Waals adduct (vdW) that must overcome a small barrier (TS vdW-INT1) to rearrange into INT1-t. This portion of the PES is crucial for determining the reaction rate coefficient. The energy values obtained in our calculations are in line with those of previous theoretical evaluations, falling within the uncertainty of the employed methods. Since the entrance channel has already been the subject of dedicated calculations,^74,76^ we did not devote additional effort to this part of the PES, considering that our CMB experiments were conducted at an *E*_*c*_ value that is quite high. All the factors that control the *k*(*T*) values have already been discussed at length in refs. 74,76 . The dissociation of INT1-t into CN + CCHCN (P1B) was also considered; however, the enthalpy change is so high ( $\Delta H_{P1B,0}^{◦}=+299.4\mathrm{kJ}/\mathrm{mol}$ relative to the reactants) that we can consider it as not accessible under most conditions of interest. The addition on the C–C triple bond can also lead to INT2 if the entering CN group attaches to the carbon already bound to a cyano group in the HC_3_N coreactant. INT2 can only dissociate into H + CC­(CN)_2_ (P2) in a very endothermic channel $\left(\Delta H_{P2,0}^{◦}=+165.5\right)$ which is, therefore, not open under most conditions of interest. *(2)* The CN radical can also add to the C atom of the HC_3_N cyano group, leading to the INT3 intermediate. In this case, a significant entrance barrier (TS R-INT3) of 21.2 kJ/mol is present. The breaking of the H–C bond of INT3 leads to the formation of a cyclic CC­(N)­CCN isomer in a very endothermic channel $\left(\Delta H_{P3A,0}^{◦}=+350.6\mathrm{kJ}/\mathrm{mol}\right)$ , while the C–C σ bond fission leads to NCCN + HCC (P3B), another endothermic channel with $\Delta H_{P3B,0}^{◦}=+54.5\mathrm{kJ}/\mathrm{mol}$ . An exit barrier associated with TS INT3-P3B (located 74.2 kJ/mol above the energy of the reactants) must be overcome. *(3)* The last addition mechanism is the one in which the CN radical adds to the N atom of HC_3_N, leading, in a barrierless process, to the formation of INT4. However, INT4 can only dissociate to CCCNCN + H (P4A) and CNCN + HCC (P4B) products in two endothermic channels ( $\Delta H_{P4A,0}^{◦}=+205.2\mathrm{kJ}/\mathrm{mol}$ and $\Delta H_{P4B,0}^{◦}=+155.8\mathrm{kJ}/\mathrm{mol}$ .

The H-abstraction channel leading to HCN + C_3_N (P9) is endothermic by 48.2 kJ/mol.

Unlike many other CN reactions with unsaturated hydrocarbons,^78,112,113^ the attacks of CN on the N-side are characterized by an entrance barrier for all possible addition sites. More specifically, *(4)* the addition to the triple C–C bond occurs with an entrance barrier of 27.9 kJ/mol and leads to INT5 in its *cis* and *trans* form (more stable than the reactants by ca. 137 kJ/mol);[Fn fn2] INT5 can dissociate into CNCCCN + H (P5A) through TS INT5-P5A (located at +64.9 kJ/mol at the CBS level of calculations), an endothermic channel with $\Delta H_{P5A,0}^{◦}=+42.6\mathrm{kJ}/\mathrm{mol}$ . INT5 can also undergo isomerization to INT8 (by overcoming a barrier located at +73.2 kJ/mol with respect to the energy asymptote of the reactants) that, in turn, can dissociate into CNCCCN + H (P5A) or CN + HCCNC (P6B) in a very endothermic channel $\left(\Delta H_{P6B,0}^{◦}=+109.7\mathrm{kJ}/\mathrm{mol}\right)$ . Alternatively, the addition of the C–C triple bond can also lead to INT6, in which the new CN group is attached to the carbon already bound to a cyano group. In this case, the entrance barrier is even higher (+ 50.4, TS R-INT6), while INT6 can dissociate into CC­(CN)­(NC) + H (P6A) products $\left(\Delta H_{P6A,0}^{◦}=+272.7\mathrm{kJ}/\mathrm{mol}\right)$ or the already cited CN + HCCNC (P6B) products. *(5)* Furthermore, the CN addition on the N-side can involve the C atom of the CN group of cyanoacetylene, but the entrance barrier is very high (+ 69.4 kJ/mol, TS R-INT7), and the addition intermediate INT7 can only dissociate into very high-energy fragments, namely the channel CNCN + HCC (P4B) and cyclic-CC­(N)­CNC + H (P7) $\left(\Delta H_{P7,0}^{◦}=+434.2\mathrm{kJ}/\mathrm{mol}\right)$ .

In the case of CN approaching from the N-side, the H-abstraction leads to products (C_3_N + HNC) in a very endothermic channel (+107.9 kJ/mol) and through a barrier of 144.7 kJ/mol.

The present results are in line with the previous calculations by Petrie and Osamura^77^ who used the CCSD­(T)/aug-cc-pVXZ//B3-LYP/6–311G** (X) D,T) levels of theory to derive the PES for the C_3_N + HNC reaction, which has some common points with the PES investigated here. For instance, from their PES, an enthalpy change of −58.6 kJ/mol can be inferred for the CN + HC_3_N → NC_4_N + H reaction, in good agreement with our value of −64.4 kJ/mol at the CBS level. The energy content of INT1-t (int_6_3 in their scheme of Figure 3 is −231.1 kJ/mol, which can be compared with our CBS value of −232.8 kJ/mol. The equivalent of our TS INT1t-P1A falls at −39.5 kJ/mol. Their geometries are also very similar.

To summarize, the evaluation of the energetic profile of the global PES reveals that only one exothermic channel is open, namely, the one that leads to dicyanoacetylene via an H-displacement mechanism. All of the other channels are endothermic, and some of them are also characterized by high energy barriers.

## 4 Discussion

The CMB data clearly suggest that an H-displacement channel leading to the formation of molecular products with a C_4_N_2_ gross formula is open. The experimental determination of the product energy release is fully consistent with the formation of dicyanoacetylene. The absence of any structure in both the LAB distributions and the best-fit CM functions indicates that no high-energy additional channels are open. Considering the energetics of the other possible H-displacement channels, as they result from the present electronic structure calculations, only the channel leading to 3-isocyano-2-propynenitrile (P5A) is barely accessible (by *ca*. 2 kJ/mol) under our experimental conditions (without considering the entrance barrier). Were this channel were to occur, features in the LAB distributions would be clearly visible due to the strong enhancement provided by the CM→LAB Jacobian to products with low CM speeds (see Figure S1). In similar cases,^79,114–116^ contributions accounting for 5% of the total yield were clearly identified and separated during the data analysis. On the contrary, here a single-contribution fit with a backward-forward symmetric T­(θ) and a smooth bell-shaped P­(E’_T_) nicely reproduces all the experimental observables.

The experimental results are perfectly in line with the main characteristics of the PES minimum energy path. The formation of stable intermediates (*cis*/*trans* INT1) with a deep potential aligns well with the backward-forward symmetric CM angular distribution, which implies the formation of a long-lived complex. The barrier on the exit channels from *cis*/*trans* INT1 has a height of 21 kJ/mol, perfectly consistent with the position of the peak of the best-fit P­(E’_T_) at 19 kJ/mol.

Concerning the possible role of the internal excitation of CN on the scattering properties, as in previous studies from our laboratory,^78,79,85^ as well as kinetics experiments^110,111^ on similar systems, the vibrational excitation of CN was not seen to promote the reaction and was not converted into product translational energy but was retained as vibrational excitation of the molecular products.

The rotational excitation of CN could affect the reaction as it was previously observed for the related CN + C_2_H_4_ reaction.^114^ In that case, it was speculated that CN rotational excitation enhances the yield of the CH_2_CHNC channel over that of the favored CH_2_CHCN one. However, unlike that case, the formation of 3-isocyano-2-propynenitrile (P5A) is strongly unfavored due to the large endothermicity and high barrier in the exit channel (TS INT5-P5A).

The results obtained in this study can be compared with the data reported in the literature for similar systems and, in particular, with the reaction CN + C_2_H_2_ since cyanoacetylene can be regarded as a functionalized acetylene with a nitrile group in place of one of the two hydrogen atoms.^82,112^ Such a comparison is particularly appropriate when carried out with the work by Casavecchia et al.^82^ and Leonori et al.,^85^ given the fact that the CN + C_2_H_2_ reaction was investigated using the same CMB apparatus, CN beam, and at a very similar collision energy of E_*c*_ = 48.1 kJ/mol. For the PES comparison, we can refer to the work by Huang et al.^112^ Starting from the experiments, the CM angular distributions are clearly different: in the case of CN + C_2_H_2_, the best-fit T­(θ) shows some forward bias, indicating the shortening of the intermediate lifetime, while the T­(θ) of the present determination is fully backward-forward symmetric. This is well explained by considering the reduced exothermicity of the channel leading to NC_4_N + H with respect to the analogous channel leading to HC_3_N $\left({\Delta H}_{0}^{◦}=-94\mathrm{kJ}/\mathrm{mol}\right)$ and the much higher exit barrier (21 kJ/mol with respect to the products, compared with +7 kJ/mol for CN + C_2_H_2_ for the most favorable channel). In addition, the presence of an additional CN group in the molecular skeleton of the reactant results in an increased number of degrees of freedom among which to distribute the large amount of energy liberated by the formation of the bound intermediates (ca. 230–240 kJ/mol in both cases).[Fn fn3]

Also, the shape of the best-fit P­(E’_T_) is quite different: in the case of the CN + C_2_H_2_, the peak is flat from E’_T_ = 20–60 kJ/mol. This characteristic suggests that two mechanisms actually contribute to the reactive signal. The second contribution was, indeed, associated with the formation of isocyanoacetylene + H, a channel that is accessible under the conditions of the experiment by Leonori et al.^85^

The similarities and differences in the PES of CN + C_2_H_2_ vs CN + HC_3_N do not only affect the reaction dynamics, but have a direct impact on the reaction kinetics as well. As already noted by Halpern et al.,^72^ at room temperature, the CN + HC_3_N reaction is significantly slower than the CN + C_2_H_2_ reaction. We now know that this is not caused by the presence of an entrance barrier, but rather associated with the effect of a vdW complex in the entrance channel and the submerged transition state vdW-INT1.^74,76^ Furthermore, we note that the addition of CN to the triple C–C bond on the N-side is barrierless in the case of the CN + C_2_H_2_ reaction. The HC=CH—NC intermediate easily isomerizes to the HC=CH—CN intermediate, which then dissociates into HC_3_N + H. In the title reaction, instead, the N-side addition reactive flux is lost in a repulsive channel.

When comparing the present results with the related CN + C_2_H_3_CN reaction, which was also investigated in our laboratory using the same experimental and theoretical approaches,^114^ some similarities can be noted. Also in this case, there was no evidence that the vibrational excitation of the CN radicals is converted into product translational energy, and the isocyano products are not formed (their formation channels are endothermic and characterized by high exit barriers). However, a small yield of 1,1-dicyanoethylene was observed in an exothermic channel characterized by submerged transition states. This is in contrast to the current system, where the equivalent path (INT2 → TS INT2-P2 → CC­(CN)_2_ + H) is strongly endothermic.

A final comparison should be made with the CN + HCN reaction since cyanopolyynes are often compared to hydrogen cyanide. The CN + HCN reaction has been studied using experimental and theoretical methods.^66,77,117,118^ Experimental studies of chemical kinetics consistently indicate the presence of a significant activation energy.^117,118^ The presence of a barrier in the entrance channel was confirmed by theoretical calculations,^66,77,117^ even when considering the favored attack, i.e., the addition of the CN radical to the carbon atom of HCN.^77^ Therefore, the situation is very different from that of the reaction under consideration. The reason for this is the greater strength of the C–N triple bond compared to the C–C triple bond, which is due to the electronegativity of nitrogen and its smaller size, enhancing orbital overlap. These effects can be easily appreciated by observing the structure of cyanoacetylene (see Figure 4), in which the C≡C bond distance is 1.201 Å (1.2 Å is the typical value for acetylenic compounds), while the C≡N distance is 1.155 Å (1.15–1.16 Å are the typical values for nitriles). This is reflected in their differing reactivity in bimolecular reactions: while it is known that CN, or other radicals, readily add to triple C–C bonds (e.g., CN + C_2_H_2_, CH_3_CCH),^41,49,50^ the cyano group present in nitriles is rarely involved in reactions that cause its breaking and is preserved in the products, even in the case of reactions with very reactive species like atomic nitrogen in the ^2^D electronically excited state (e.g., N­(^2^D) + CH_3_CN, N­(^2^D) + C_2_H_3_CN, and N­(^2^D) + HC_3_N).^89,114,119,120^ The characteristics of the cyano group in nitriles are so unique that cyano is considered a pseudohalogen.

## 5 Implications for Astrochemistry and Cosmochemistry

Our work presents experimental evidence that NC_4_N is the sole molecular product formed in the only open H-displacement channel. 3-Isocyano-2-propynenitrile is not formed with a measurable yield within the sensitivity of our technique, even though the total energy available to the reactants, when considering the collision energy and the internal energy of the CN radical, is sufficient to overcome the exit barrier and to compensate for the positive enthalpy change associated with that channel. The other possible reaction channels are even more endothermic and are not seen to occur under the conditions in our experiments. It is not straightforward to convert the collision energy of CMB experiments into temperature. Based on previous work, it can be inferred that the collision energy of this experiment corresponds to a temperature between 300 and 1000 K.^121,122^ Obviously, at the low temperatures of relevance to the atmosphere of Titan or cold interstellar regions, NC_4_N + H is the only possible set of products.

After the determination of the rate coefficient at low temperature, the title reaction has been considered in the photochemical models of the atmosphere of Titan as a possible formation route of dicyanoacetylene, together with other, less-characterized reactions (e.g., N + HC_4_N or C_3_N + HCN).^52,53^ However, photochemical models still underpredict the amount of NC_4_N and the related species C_2_N_2_,^52,53^ pointing to the fact that the chemistry of these two species has not yet been well understood.

On the contrary, the CN + HC_3_N reaction is not mentioned as a possible formation route of NC_4_N in TMC-1 by Agúndez et al.^64^ Agúndez et al.^64^ were only referring to the reactions proposed by Petrie and Osamura^77^ and Petrie et al.^66^ for C_2_N_2_ and NC_4_N.

5 $$C_{3}N+\mathrm{HNC}\to {\mathrm{NC}}_{4}N+H$$

6 CN+HNC→NCCN+H

However, considering the large fractional abundance of both CN and HC_3_N in TMC-1 (both in the order of 10^–8^)^40^ and the large rate coefficient measured at low T, the title reaction is clearly an important formation route of dicyanoacetylene that should be considered in astrochemical models. In the recent upgraded version of the UMIST Database for Astrochemistry, the CN + HC_3_N reaction has been included in both the TMC-1 and IRC+10216 models using the fit expression of the CRESU data^74^ and assuming that NC_4_N + H is the sole reaction channel. The resulting simulated fractional abundance of NC_4_N^+^ calculated at 1.6 × 10^5^ years for the O-rich model or at 1.0 × 10^6^ years for the C-rich model is in good agreement with the value derived by Agúndez et al.^64^

Interestingly, the title reaction is considered a termination step in the sequence of reactions that lead to the formation of long cyanopolyynes (the largest observed one is HC_11_N)^123^ in the interstellar medium and circumstellar envelopes (see Cheikh Sid Ely et al.^74^ and references therein):

7 $$C_{2}H+H-(C≡{C-)}_{n}H\to H-(C≡{C-)}_{n+1}H+H$$

8 $$\mathrm{CN}+H-(C≡{C-)}_{n}H\to H-(C≡{C-)}_{n}\mathrm{CN}+H$$

9 $$C_{2}H+H-(C≡{C-)}_{n}\mathrm{CN}\to H-(C≡{C-)}_{n+1}\mathrm{CN}+H$$

10 $$\mathrm{CN}+H-(C≡{C-)}_{n}\mathrm{CN}\to \mathrm{NC}-(C≡{C-)}_{n}\mathrm{CN}+H$$

To verify whether our results for the title reaction can be extended to other reactions in the CN + H–(C≡C−)_*n*_CN series, we performed calculations for the CN + HC_5_N reaction for the minimum energy pathway only at the CCSD (T)//B3LYP/aug-cc-pVTZ and CBS levels of theory (determining the full PES, as presented in Figures 7 and 8F require dedicated work and a separate publication). Again, we did not find an entrance barrier above the energy of the reactants; the reactants correlate with a covalent intermediate (INT9 inFigure [Fig fig9]), and its dissociation into the NC_6_N + H products proceeds with a barrier that remains well below the energy of the reactants. Therefore, this reaction will be characterized by a rate coefficient in the gas kinetics limit (in the 10^–10^ cm^3^ s^–1^ range) even at the very low temperatures of interest in the ISM or the atmosphere of Titan.

**Figure 9:**
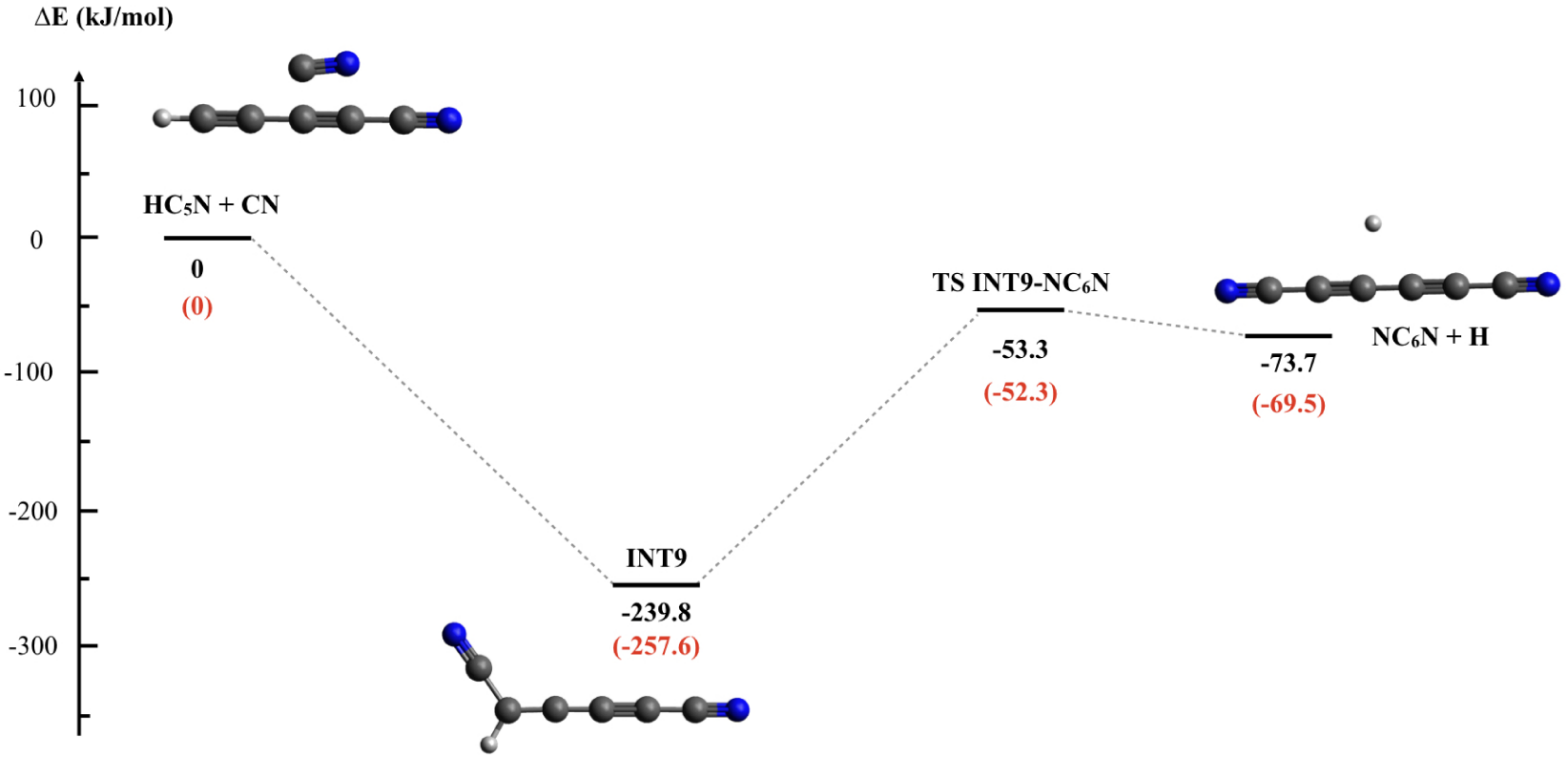
Minimum energy path in the potential energy surface for the CN + HC_5_N reaction. Energies are computed in kJ/mol at the CCSD­(T)//B3LYP/aug-cc-pVTZ (black) and CBS (red) levels.

The formation of NC—(C≡C—)_*n*_CN (including dicyanoacetylene with *n* = 1) stops further growth in the eqs 7–10 chain since dicyanopolyynes are unlikely to be reactive toward further attack by CN or C_2_H, as suggested by Cheikh Sid Ely et al.,^74^ who mentioned the low rate coefficient for the reaction CN + NC_4_N at room temperature (5.4 × 10^–13^ cm^3^ s^–1^)^124^ and their preliminary calculations (unpublished to the best of our knowledge) on the entrance barrier of CN + NC_4_N and C_2_H + NC_4_N. Since this aspect is crucial to understanding the growth and destruction routes of cyanopolyynes, we have also carried out calculations for the entrance channel of the last reaction. As visible in Figure 10, all of the possible addition sites of C_2_H to NC_4_N are indeed characterized by a significant entrance barrier that would make this reaction impossible in the low T regions of the interstellar medium. However, this does not necessarily mean that an environment with abundant CN radicals will impede the growth of cyanopolyynes by converting them into dicyanopolyynes: a recent astrochemical model revealed that HC_5_N is formed essentially by the reactions N + C_6_H, C_2_H + HC_3_N, C_3_N + C_2_H_2_, and C_4_H + HCN in cold clouds and C_3_N + C_2_H_2_ in shocked regions.^39^ In the same work, the CRESU rate coefficients for the reactions C_2_H + HC_3_N and C_3_N + C_2_H_2_ were provided (for temperatures as low as 24 K), as well as the potential energy surface with a kinetic analysis for three other possible formation routes of HC_5_N (N + C_6_H, C_4_H + HCN, and C_4_H + HNC). Therefore, the importance of reaction (8) in the formation of larger cyanopolyynes seems to be reduced, at least in the case of HC_5_N, but larger cyanopolyynes could be formed in similar ways.

**Figure 10:**
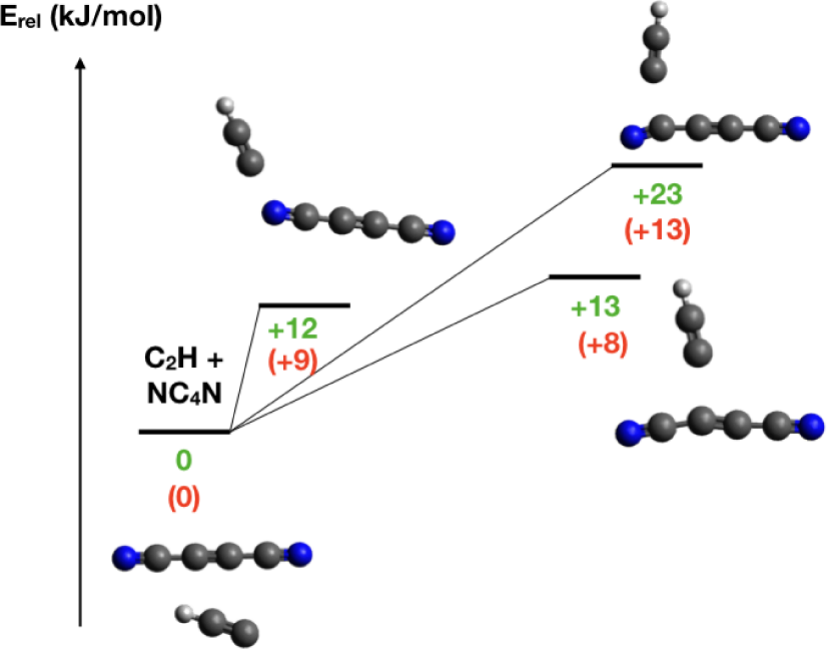
Entrance channels of the potential energy surface for C_2_H + NC_4_N. Energies (in kJ/mol) are in green at the CCSD­(T)/aug-cc-pVTZ//M062X/6-311+G­(d,p) level and in red at the CBS level.

From the global PES that we have derived in this work, we can also infer some properties of other reactions that have not been investigated previously (to the best of our knowledge). For instance, the reaction C_2_H + C_2_N_2_ is characterized by a significant entrance barrier (+ 19.7 kJ/mol), and, therefore, it cannot occur either in the interstellar medium or in the atmosphere of Titan. On the contrary, the C_2_H + CNCN reaction is barrierless and easily produces HC_3_N + CN. It can therefore be considered either a destruction route of isocyanogen or an alternative formation route of HC_3_N (provided that isocyanogen is available).

Finally, it is worth noticing that the reaction CN + isocyanoacetylene (P6B in the scheme of Figure 8) is barrierless in the entrance channel and can easily form both 3-isocyano-2-propynenitrile + H or cyanoacetylene + CN according to the sequences:

- CN + HCCNC (isocyanoacetylene) → INT8 (−246 kJ/mol) → TS INT8-P5A (−43.2 kJ/mol) → CNC_3_N (3-isocyano-2-propynenitrile) + H (−72.1 kJ/mol)
- CN + HCCNC (isocyanoacetylene) → INT6 (−211.1 kJ/mol) → TS R-INT6 (−59.3 kJ/mol) → CN + HC_3_N (−109.7 kJ/mol)

where the energy values are those calculated at the CCSD­(T)//B3LYP/aug-cc-pVTZ level of theory and assuming the energy content of CN + HCCNC as the zero of the energy scale. Since TS R-INT6 is lower in energy than TS INT8-P5A, the formation of cyanoacetylene + CN is favored. In other words, the reaction of CN with isocyanoacetylene is an efficient way to convert it to cyanoacetylene with a null consumption of CN radicals. This can be generalized for all reactions of the type CN + isocyanopolyynes, a new class of reactions that could help better understand the relationship between the abundance of cyanopolyynes and isocyanopolyynes. For instance, in TMC-1 [HC_3_N]/[HCCNC] is 77.0 ± 8.0, while in IRC+10216, it is 392 ± 22.^125^ Considering the next member of the series, [HC_5_N]/[HC_4_NC] is 600 ± 70 in TMC-1 and ≥ 2000 in IRC+10216.^125^ The origin of the large difference between the TMC-1 and IRC+10216 values is not yet known nor is the variation along the series.

We recommend testing the conversion of isocyanopolyynes into their cyano counterparts through reactions with CN in astrochemical models. In CN-rich environments, we expect that the cyanopolyynes are converted into dicyanopolyynes, but at the same time, isocyanopolyynes are mostly converted into cyanopolyynes. The role of these two contrasting trends can only be assessed in astrochemical models. A recent model proposed by Xue et al.^126^ for TMC-1 overproduces both HCCNC and HC_4_NC. In that model, an important destruction route for both cyanopolyynes and isocyanopolyynes is the reaction with atomic carbon, which is considered to occur with the same efficiency in both cases (see also Loison et al.^40^ and Li et al.^127^). Since the CN radicals can be as abundant as C atoms, the reactions of cyanopolyynes and isocyanopolyynes are expected to be important destruction routes but with a very different outcome. For instance, the increased fractional abundance of CN radicals in the CSEs of AGB stars (such as IRC+10216) of at least 1 order of magnitude wrt TMC-1 ^22^ could explain why isocyanopolyynes are almost absent, even though their formation routes are the same as those considered for TMC-1.

## 6 Conclusions

In this work, we present an experimental and theoretical study of the reaction between the cyano radical and cyanoacetylene. Based on experimental evidence and electronic structure calculations of the relevant potential energy surface, the reaction involving cyanoacetylene and the cyano radical leads to only one exothermic channel associated with the formation of dicyanoacetylene. Furthermore, we derive some properties of the related reactions C_2_H + CNCN (isocyanogen) and CN + HCCNC (isocyanoacetylene): the C_2_H + CNCN reaction leads to the formation of CN + HC_3_N, and the main channel of the CN + HCCNC reaction also leads to CN + HC_3_N. This last reaction efficiently converts isocyanoacetylene and, by extension, any isocyanopolyyne into their cyano counterparts without a net loss of cyano radicals. The effects of this new family of reactions should be tested in astrochemical models.

Finally, we also characterized the CN + HC_5_N reaction (confirming that it easily evolves toward NC_6_N + H) and the entrance channel of the reaction C_2_H + NC_4_N. The addition of C_2_H to all possible sites of NC_4_N is characterized by a significant entrance barrier, thus confirming that, once formed, dicyanoacetylene terminates the growth of cyanopolyynes.

## Supplementary Material



## Footnotes

1 NIST Mass Spectrometry Data Center Collection (C) 2014, copyright by the U.S. Secretary of Commerce on behalf of the United States of America. All rights reserved.

2 For clarity, only the *cis* isomer is shown in Figure 8.

3 In the reaction, a second intermediate can be formed with the structure CH_2_CCN via a 1,2 H-shift; this channel gives a sizable contribution to the reaction. An analogous process for the title reaction would require a much more complex CN-shift and does not occur.

## Notes

Simulation of the laboratory distributions using CM functions for the CNC_3_N + H channel T1 analysis (PDF)

## #

CEA, DES, ISEC, DMRC, Univ. Montpellier, Marcoule, France
