# Supplementary material for: Dicyanoacetylene (NC4N) Formation in the CN + Cyanoacetylene (HC3N) Reaction: A Combined Crossed-Molecular Beams and Theoretical Study
Source: ACS Earth Space Chem. 2025 Jul 31;9(8):2199–214. doi: 10.1021/acsearthspacechem.5c00154 (PMC12376185; doi:10.1021/acsearthspacechem.5c00154)

Dicyanoacetylene (NC_4N) formation in the CN + cyanoacetylene (HC_3N) reaction: a combined crossed molecular beams and theoretical study

Emilia Valenca Ferreira de Aragao,[†,‡,§] Pengxiao Liang,[†] Luca Mancini,[†,‡]Gianmarco Vanuzzo,[†,‡] Giacomo Pannacci,[†] Noelia Faginas-Lago,[†] Piergiorgio Casavecchia,[†] Marzio Rosi,[¶] and Nadia Balucani[*,†]

[†]*Dipartimento di Chimica, Biologia e Biotecnologie, Universit`a degli Studi di Perugia, Via Elce di Sotto, 8, 06123, Perugia, Italy*
[‡]*Master-Tec S.r.l., Via Gerardo Dottori, 94, 06132 Perugia, Italy*
[¶]*Dipartimento di Ingegneria Civile ed Ambientale, Universit`a degli Studi di Perugia, Via G. Duranti, Perugia, Italy*
[§]*Current address: CEA, DES, ISEC, DMRC, Univ Montpellier, Marcoule, France*

E-mail: nadia.balucani@unipg.it

Table S1: T1 diagnostic values for all the stationary points included in the minimum energy path (both the C-side and N-side addition) of the reaction CN + HC_3N.

Stationary Point	T1 Diagnostic Factor
vdW	0.029
TS vdW-INT1	0.021
INT1-t	0.022
TS c-t	0.020
INT1-c	0.021
TS INT1t-P1A	0.016
TS INT1c-P1A	0.016
TS R-INT5	0.030
INT5	0.022
TS INT5-P5A	0.018

Figure S1: Simulation of the laboratory angular (a) and time-of-flight (b-d) distributions when assuming that the CNC_3N product is formed in the H-displacement channel (canonical Newton diagram). We have used an isotropic CM angular distribution and the P(E'$_T$) shown in panel (e) corresponding to a fraction of the total available energy of 0.33 (a quite typical value). Given the small amount of the available energy (this reaction channel is endothermic by 42.6 kJ/mol and the collision energy is 44.8 kJ/mol) the simulated angular distribution is quite sharp. The only simulated time-of-flight distribution that is not null is the one at the angle close to Θ_{CM}=30.6°. However, the simulated distribution is too sharp around the flight time corresponding to the center-of-mass velocity.

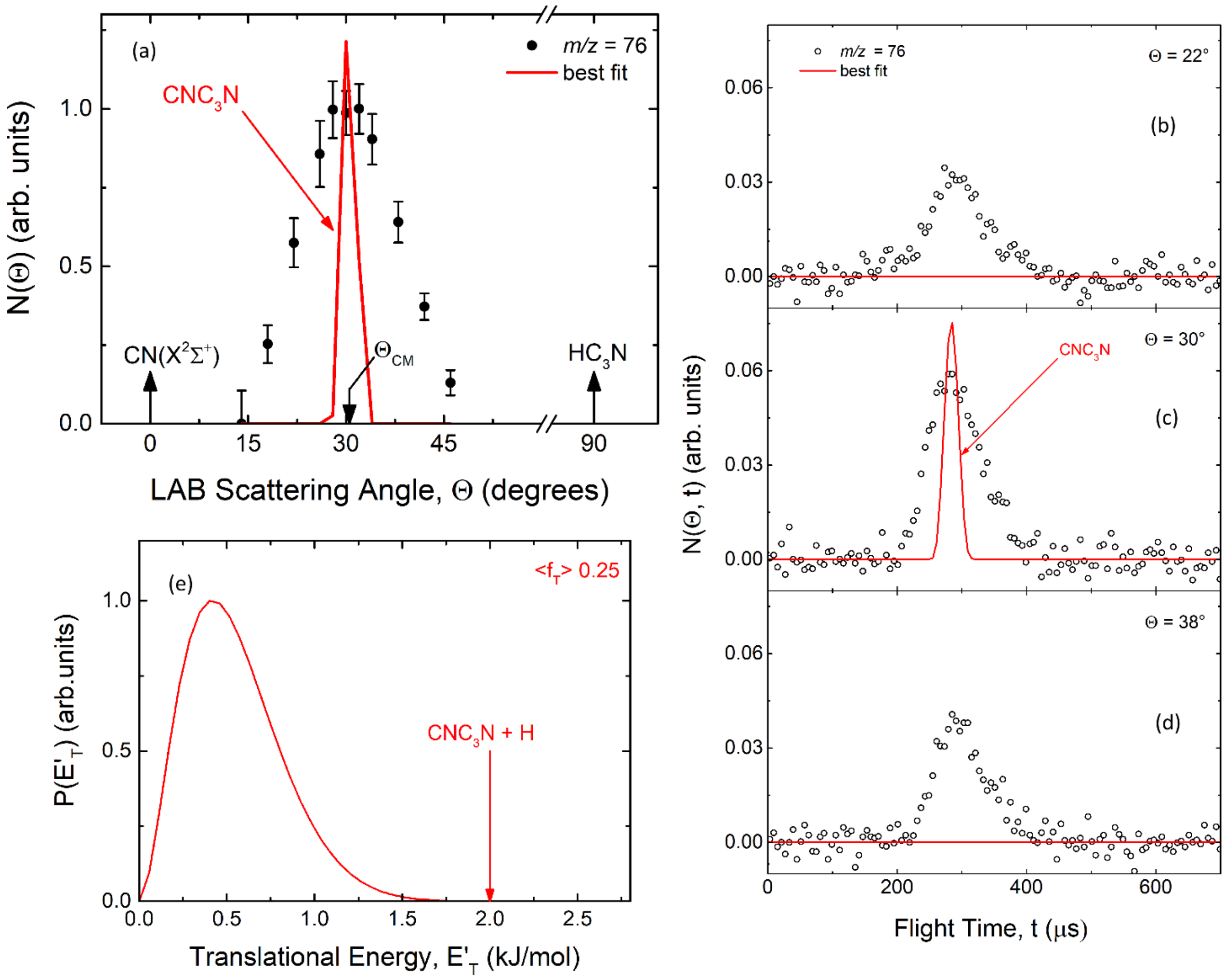

Supplement: Supplementary file 1 [file sp5c00154_si_001.pdf]
